# Multimodal imaging findings of multiple evanescent white dot syndrome in COVID-19 patients

**DOI:** 10.1016/j.idcr.2024.e02110

**Published:** 2024-11-03

**Authors:** Natalie Chen, Mark Mandell, Parnian Arjmand

**Affiliations:** a Temerty Faculty of Medicine, University of Toronto, Toronto, Ontario, Canada; b Department of Ophthalmology and Vision Sciences, University of Toronto, Toronto, Ontario, Canada; c Mississauga Retina Institute, Mississauga, Ontario, Canada; d Department of Ophthalmology, St. Michael’s Hospital, Toronto, Ontario, Canada; e Baycrest Health Sciences Centre, Toronto, Ontario, Canada

**Keywords:** Multiple evanescent white dot syndrome, White dot syndromes, Infectious diseases, COVID-19, Retinal diseases

## Abstract

**Purpose:**

To describe the multimodal imaging findings of a rare case of multiple evanescent white dot syndrome (MEWDS) associated with COVID-19.

**Methods:**

A case report was analyzed and described alongside COVID-19 associated MEWDS cases identified in the current literature.

**Results:**

A healthy 20-year-old man was evaluated after a three-day history of blurry vision occurring two months after COVID-19 infection. Multimodal imaging revealed signs of typical MEWDS, with optical coherence tomography angiography (OCT-A) demonstrating homogenous reflectivity. Six additional cases were reported in the literature, displaying clinical symptoms and imaging consistent with typical MEWDS but demonstrating higher rates of incomplete visual recovery and treatment use.

**Conclusions:**

COVID-19 associated MEWDS is a novel condition. This is the first known case of COVID-19 associated MEWDS with reported OCT-A findings in an otherwise healthy patient. Although posterior uveitis following COVID-19 infection is rare, clinicians should remain informed on the best practices for diagnosing and caring for patients with MEWDS.

## Introduction

Coronavirus disease 2019 (also known as COVID-19) is a highly infectious illness caused by the SARS-CoV-2 coronavirus, with common symptoms including fever, fatigue, cough, and dyspnea [1]. While commonly thought of as a respiratory condition, COVID-19 can cause multi-system disease due to its affinity for angiotensin-converting enzyme 2 (ACE2) receptors located throughout the body [2]. One common site of infection is the eyes, with conjunctivitis being the most frequently documented ophthalmic manifestation [3]. While the virus has less frequent manifestations in the ophthalmic posterior segment, entities such as vitritis and choroiditis have all been reported in COVID patients [3].

Multiple evanescent white dot syndrome (MEWDS) is a rare white dot syndrome that most frequently manifests as unilateral posterior uveitis [4]. It typically affects individuals between ages 20–40 years, with propensity towards women and those with a history of myopia [4]. Common clinical symptoms include an acute decrease in visual acuity, photopsias, and an enlarged blind spot on visual field testing [4]. Clinically, MEWDS is characterized by fine outer retinal foveal granular changes as well as numerous pale and creamy chorioretinal lesions around the optic nerve or spread throughout the posterior pole and/or mid-periphery [4]. These chorioretinal lesions, which are more clearly seen on fundus autofluorescent imaging, may resolve rapidly and can be missed in patients who present in the subacute phase of the disease. Other associated features include the presence of disc edema and vitritis [5]. Up to 50 % of MEWDS cases have been preceded by a viral infection [4]. While no clear pathogenesis has been found, some studies have attributed MEWDS infection to an autoimmune response secondary to the viral invasion [6]. There is limited literature reporting COVID-19-associated MEWDS cases. Here, we describe multimodal imaging findings in a pathognomonic case of MEWDS following an active COVID infection. We further describe all cases of COVID-associated MEWDS cases published between December 2019 to June 2024 on OVID Medline, PubMed and Cochrane that were identified using the search strategy: ("white dot syndrome" OR "multiple evanescent white dot syndrome") AND ("COVID-19" OR “COVID-19 vaccin*”).

## Case report

A 20-year-old otherwise healthy man presented with a 3-day history of blurry vision in the left eye that started approximately two months following a COVID-19 infection confirmed by a home antigen test. Past medical and ocular history were unremarkable. The review of symptoms was overall negative for any skin, neurologic, respiratory or gastrointestinal symptoms, and there was no history of sexually transmitted infections or intravenous drug use. There was also no history of exposure to animals, tuberculosis (TB), or consumption of raw meats. A posterior uveitis work-up was completed and demonstrated a normal complete blood count (CBC); normal ACE, ANA and RF levels; as well as negative serology for toxoplasmosis, syphilis, and *bartonella henselae*. Chest X-ray and TB skin test were also negative.

**Fig. 1:**
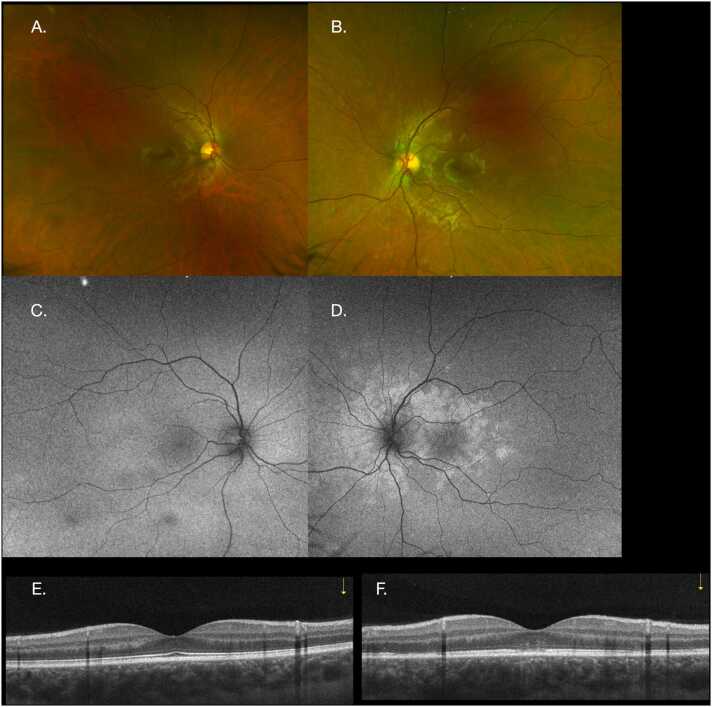
Color fundus photo of the right eye is within normal limits (A) but shows scattered small peripapillary creamy retinal lesions in the left eye (B). Fundus autofluorescence imaging (FAF) was normal in the right eye (C) but showed a diffuse hyperautofluorescent pattern along the posterior pole corresponding to retinal lesions in the left eye (D). Optical coherence tomography (OCT) was normal in the right eye (E) but shows outer retinal attenuation and RPE disruption in the left eye (F).

**Fig. 2:**
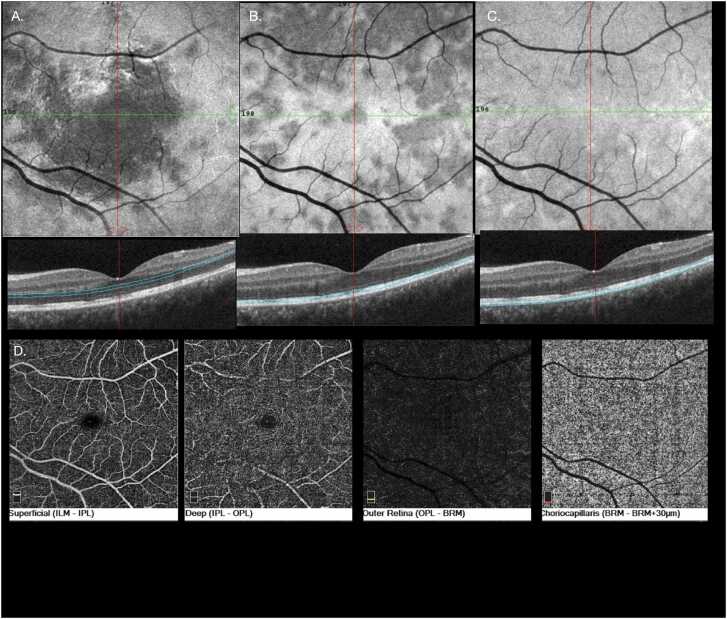
En-face images at the levels of the outer plexiform/outer nuclear layers in the left eye (A), external limiting zone and ellipsoid (B), and retinal pigment epithelium (RPE) (C) show hyporeflective lesions corresponding to areas of attenuation on OCT B scans. OCT angiography (OCTA) of the left eye shows homogenous reflectivity with no transmission defects or artefacts (D).

On examination, the best corrected visual acuity (BCVA, Snellen chart) was 20/20 in each eye and the anterior segment exam was within normal limits. A dilated fundus exam was normal in the right eye (RE) (Fig. 1A), and showed scattered, small, peripapillary creamy white retinal lesions in the left eye (LE) (Fig. 1B). Fundus autofluorescence (FAF) imaging was also normal in the RE (Fig. 1C) but showed a diffuse hyperautofluorescent pattern along the posterior pole corresponding to retinal lesions in the LE (Fig. 1D). Optical Coherence Tomography (OCT) was normal in the RE (Fig. 1E) and showed attenuation and granularity along the external limiting membrane (ELM), ellipsoid layer, and retinal pigment layer (RPE) disruption in the LE (Fig. 1F). En-face scans at the level of the outer plexiform/outer nuclear (Fig. 2A), outer retinal (ELM and ellipsoid) (Fig. 2B) and RPE (Fig. 2C) demonstrated hyporeflective lesions corresponding to areas of attenuation on OCT B scan. OCT Angiography (OCT-A) for the LE also shows homogenous reflectivity with no transmission defects or artefacts (Fig. 2D).

The patient’s clinical presentation, examination findings, and imaging were consistent with the diagnosis of MEWDS. Due to the self-limiting nature of this entity, no treatment was initiated. The patient felt subjectively better after three days and had 20/20 vision at 3 months’ follow-up. Ethical approval was not sought as this was a case report, and written informed consent was obtained from the patient regarding the publication of patient information and images.

## Discussion

Here, we report a case of MEWDS after confirmed COVID-19 infection in an otherwise healthy patient, present multimodal imaging findings, and discuss all reported cases of MEWDS related to COVID-19 infection, exposure, or vaccination in the current literature. To the best of our knowledge, this is the first reported MEWDS case in an otherwise healthy patient following a confirmed COVID-19 infection with reported OCT-A findings.

MEWDS is an ocular inflammatory syndrome that is often preceded by a viral illness such as adenovirus-related upper respiratory tract infections (URTIs) [4]. During the COVID pandemic between March 2020 to March 2022, an uptick in several inflammatory entities was reported across multiple medical specialties [7], [8]. From an ocular inflammation and uveitis standpoint, a significant spike in all reported MEWDS cases by 0.02 % was reported between March 2020 to March 2023 compared to the pre-pandemic incidence of this condition [9]. Furthermore, six other case reports in addition to our study have described MEWDS in confirmed COVID-19 patients (Table 1) [10], [11], [12], [13], [14], [15]. The average age for these patients was 38.4 years (with a range of 17 to 69 years) and five of seven patients were female, in keeping with the known demographic distribution affected by MEWDS. Two patients had a reported history of myopia, which is commonly associated with MEWDS. The average time from COVID-19 infection to initial MEWDS symptoms was 29.6 days but ranged between 0 to 70 days.

**Table 1:** Summary of COVID-19 associated MEWDS cases.

Authors	Age	Gender	Past Ocular History	Past Medical History	Time from infection to symptoms (days)	Eye (s) affected	Ocular Symptoms	Treatment for MEWDS	Initial VA (Snellen)	Final VA of Affected Eye (Snellen)
Gallo et al. [16]	37	F	Mild bilateral myopic refractive error.	Cutaneous melanoma with groin lymph node involvement.	0	RE	Decreased vision, central scotoma, occasional photopsias, and floaters.	Six monthly aflibercept injections for 5 months.	HM (RE), 20/20 (LE)	20/160
. Zecevic et al. [17]	40	F	Not stated.	Hypertension, Hashimoto thyroiditis.	14	LE	Vision impairment.	Nepafenac solution and acetazolamide for 1 week.	20/20 (RE), 20/200 (LE)	20/25
Jain et al. [18]	17	M	Not stated.	Not stated.	70	Both	Blurry vision, photopsias, and floaters.	Oral prednisolone for 5 months.	20/32 (RE & LE)	20/16 (RE & LE)
Ting et al. [19]	69	F	Mild bilateral nuclear cataract, myopic refractive error with mild retinal degeneration.	Asthma, hypothyroidism, and headaches.	28	LE	Floaters, central flashing, enlarging scotoma, and progressive vision loss.	Oral steroids for 4 weeks.	20/30 (RE), 20/80 (LE)	20/50
De Salvo et al. [20]	39	F	Not stated.	Postpartum depression, acephalgic migraine, and asthma.	14	LE	Floaters and progressive vision loss.	None.	20/40 (RE), 20/200 (LE)	20/50
Smeller et al. [21]	47	F	Not stated.	Simultaneous reactivation of herpes simplex virus.	21	Both	Photophobia and blurry vision (bilateral), decreased visual acuity (LE).	Corticosteroid injections for 5 times, acyclovir for 5 days, corticosteroid drops, and cycloplegicedol for an unspecified duration.	20/20 (RE), 20/100 (LE)	20/20 (both eyes)
Chen et al. (our case)	20	M	None.	None.	60	LE	Blurry vision	None.	20/20 (RE), 20/20 (LE)	20/20

Clinically, all patients exhibited symptoms consistent with MEWDS. However, two patients (29 %) presented with bilateral involvement, an unusual manifestation that has only been reported a few times in the current literature [16]. Interestingly, a 2024 systematic review describing MEWDS cases following COVID vaccination found that all included males had a bilateral presentation [17]. As one of the patients with bilateral involvement in our review was also male, further research exploring the relationship between gender and laterality in COVID-associated MEWDS cases may be warranted. Another unusual characteristic of MEWDS cases in reported COVID patients was the rate of incomplete visual recovery, as MEWDS patients typically experience full visual recovery within the following weeks or months [4]. However, one patient may not have had time to fully recover, as their follow-up examination occurred one week after treatment began. Lastly, it is important to note that five patients (71 %) received treatment for their symptoms. As MEWDS is considered a self-limiting disease, treatment options are seldomly used and reserved for cases with recurrent, prolonged or significant vision loss [13]. This suggests that COVID-19 MEWDS cases may be more severe when compared to other viral etiologies and should be further investigated.

To our knowledge, our case is one of only two published cases with OCT-A findings in MEWDS secondary to COVID and the first case outlining typical OCT-A findings in a patient post-COVID infection. OCT-A is an important imaging modality in documenting chorioretinal vasculature on presentation and follow-up in patients with white dot syndrome [18]. Gallo et al. reported OCT-A findings in a MEWDS case complicated by choroidal neovascular membrane (CNVM) in a COVID patient who was simultaneously receiving immunotherapy including dabrafenib and trametinib for skin melanoma [10]. The authors speculated that the use of immunotherapy, alongside an active COVID-19 infection, may have contributed to an inflammatory milieu and resulted in a more serious MEWDS phenotype. Specific imaging findings for all patients can be found in Table 2**.** All patients demonstrated disruption of the deep retinal layers in OCT. Fundus examination revealed white, grayish or yellowish lesions scattered throughout the posterior pole, mid- or inferior periphery, or surrounding the optic disc. Of the patients with FAF findings, five patients (71 %) demonstrated hyperautofluorescence, while one patient (14 %) had hypofluorescent lesions. Hyperfluorescent dots, often seen in a rim-like pattern, and late-stage staining were observed on fluorescein angiography (FA). Finally, three patients (43 %) received indocyanine-green angiography (ICGA). Two patients (29 %) demonstrated hypocyanescence and hypofluorescence in the posterior pole and peripapillary regions, which are typically seen in MEWDS patients. However, one patient had hypercyanescent lesions that the authors attributed to the novel and often unknown pathophysiology of COVID-19.

**Table 2:** Initial imaging findings of affected eye(s) in COVID-19 associated MEWDS cases.

Authors	Imaging Conducted	Optical Coherence Tomography (OCT) Findings	Optical Coherence Tomography Angiography (OCT-A)	Fundus Examination (FE)/Photography (FP)	Fundus Autofluorescence (FAF) Findings	Fluorescein Angiography (FA) Findings	Indocyanine-Green Angiography (ICGA)
Gallo et al. [16]	OCT, OCT-A, FE, FAF, FA	Disruption of the retinal pigment epithelium, ellipsoid zone and interdigitation zone, and external limiting membrane.	Parafoveal choroidal neovascularization.	Optic discs had blurry margins. Presence of yellow exudates at the macula, and grayish spots in the posterior pole and mid-periphery.	Foveal hypoautofluorescence that was surrounded by diffuse hyperautofluorescence observed in the posterior pole and peripapillary regions.	Hyperfluorescent spots, with early hypofluorescence and late leakage seen in the macula.	Hypofluorescent dots as well as diffuse hypofluorescence in the posterior pole and peripapillary regions.
Zecevic et al. [17]	OCT, FP, FAF, FA	Disruption of the ellipsoid zone and multifocal nodular thickening in the retinal deep layers.	Not reported.	Numerous white dots surrounding the optic disc and throughout posterior pole.	Hypofluorescent spots in the posterior pole.	Early hyperfluorescence appearing in a rim-like pattern, late stage staining in areas as indicated by white dots.	Not reported.
Jain et al. [18]	OCT, FP, FAF, FA, ICGA	Disruption of the ellipsoid zone, with decreased attenuation of the external limiting membrane.	Not reported.	Several hypopigmented choroidal lesions near the macula, inferior periphery regions, and nasal to the optic disc.	Conducted but findings not reported.	Early hyperfluorescence observed, with late staining.	Hypocyanescence observed.
Ting et al. [19]	OCT, FAF, FA	Disruption of the ellipsoid zone and hyper-reflective subretinal deposits in the LE.	Not reported.	Not reported.	Hyperautofluorescent spots in the posterior pole affecting the peripapillary and macular regions.	Punctate hyperfluorescent areas with well-defined borders in a wreath-like arrangement.	Not performed due to iodine allergy.
De Salvo et al. [20]	OCT, FE, FAF, FA, ICGA	Disruption of the ellipsoid zone and hyper-reflective material extending into the outer nuclear layer.	Not reported.	Several yellowish lesions located throughout the retina with tortuous dilated veins.	Hyperfluorescence in the outer retina and enhanced optic disc.	Hyperfluorescent lesions spread throughout the retina.	Intermediate phase hypercyanescent lesions with late-phase resolution.
Smeller et al. [21]	OCT, FE, FAF, FA	Disruption of the ellipsoid zone, with swelling of the outer layers of retina at the level of the RPE.	Not reported.	Flat, grayish-white lesions were observed in the retinal pigmental epithelium of both eyes, with the macular region also being implicated in the LE.	Hyperautofluorescent spots.	Early and late hyperfluorescence appearing in a wreath-like pattern.	Not reported.
Chen et al. (our case)	OCT, OCT-A, FE, FAF	Disruption to the external limiting membrane, ellipsoid, and RPE.	Sub-foveal granularity.	Scattered, small, peripapillary white chorioretinal lesions.	Hyperautofluorescent lesions along the posterior pole.	Not reported.	Not reported.

While COVID-19 associated MEWDS has only been recently reported in the literature, there has been significant focus on the relationship between COVID-19 vaccines and MEWDS. This may be because MEWDS was the most common form of white dot syndrome diagnosed in patients following COVID-19 vaccination [17]. A systematic review conducted by Zeng et al. analyzed 27 patients with COVID-19 vaccine associated MEWDS from 19 articles published before November 2023 [19]. Of these patients, 18 patients received their vaccine from Pfizer BioNTech, 3 from Moderna, 2 from Sinovac, 2 from Oxford AstraZeneca, 1 from Covishield, and 1 from Medigen Vaccine Biologics Corporation. The median age of patients was 34.1 years (ranging from 15–71 years), with a female predominance. The average time from vaccination to symptom onset was 14.7 days, with a range of 1 to 90 days. Common clinical symptoms included blurred vision, photopsia, scotomas and visual field defects. Eleven patients received treatment, including 6 who were prescribed oral steroids, and 18 patients experienced a full visual recovery. Another study conducted by Kashyap and colleagues reviewed 31 MEWDS cases following COVID-19 vaccination, with similar findings to Zeng et al.’s systematic review [20]. Specifically, the average age of all patients was 33.7 years, with 77.4 % being women. Commonly observed ocular symptoms were blurry vision and photopsia, with the most popular management options including close monitoring followed by oral steroids. These findings reveal that the patient demographics, presentation, treatment, and resolution of COVID-19 vaccine-associated MEWDS cases are similar to MEWDS cases associated with COVID-19 infection. However, the average times between vaccination and symptom onset reported in Kashyap et al.’s review were 7.5 days in patients receiving their first dose and 13.1 days in patients receiving their second dose. This incubation period is much shorter when compared to COVID-19-associated MEWDS cases and should be considered by clinicians during diagnosis and management.

It is important to note that no articles investigating the association between COVID-19 exposures and MEWDS were identified. This may be because MEWDS is thought to be an atypical immune response to a viral infection; therefore, an exposure (without confirmation of infection) may not be sufficient to establish reasonable causation of MEWDS. Additional research should be conducted into the pathophysiology of MEWDS and COVID-19 exposure to identify any possible relationships.

## Conclusion

MEWDS is a rare but reported cause of vision disruption in patients recovering from COVID infection. As such, physicians should be aware of this diagnosis, patient symptoms, imaging findings, and treatment options when caring for patients presenting with blurry vision and photopsias following a COVID infection.

## CRediT authorship contribution statement

**Natalie Chen:** Investigation, Methodology, Writing – original draft, Writing – review & editing. **Mark Mandell:** Conceptualization, Investigation, Resources, Writing – review & editing. **Parnian Arjmand:** Conceptualization, Investigation, Methodology, Project administration, Resources, Supervision, Writing – review & editing.

## Ethical Approval

This case report was conducted in accordance with the Declaration of Helsinski. The collection and evaluation of all protected patient health information was performed in a US Health Insurance Portability and Accountability Act (HIPAA)-compliant manner.

## Consent

Written informed consent was obtained from the patient for publication of this case report and accompanying images. A copy of the written consent is available for review by the Editor-in-Chief of this journal on request.

## Funding

This research did not receive any specific grant from funding agencies in the public, commercial, or not-for-profit sectors.

## Declaration of Competing Interest

The authors declare the following financial interests/personal relationships which may be considered as potential competing interests: Parnian Arjmand reports a relationship with Bayer Corporation that includes: consulting or advisory. Parnian Arjmand reports a relationship with F Hoffmann-La Roche Ltd that includes: consulting or advisory. Parnian Arjmand reports a relationship with Apobiologix that includes: consulting or advisory. If there are other authors, they declare that they have no known competing financial interests or personal relationships that could have appeared to influence the work reported in this paper.
